# Decoding immune cell interactions during cardiac allograft vasculopathy: insights derived from bioinformatic strategies

**DOI:** 10.3389/fcvm.2025.1568528

**Published:** 2025-04-24

**Authors:** Edward B. Thorp, Aparnaa Ananthakrishnan, Connor W. Lantz

**Affiliations:** ^1^ Department of Pathology, Feinberg School of Medicine, Northwestern University, Chicago, IL, United States; ^2^ Department of Surgery, Comprehensive Transplant Center, Feinberg School of Medicine, Northwestern University, Chicago, IL, United States

**Keywords:** immunity, bioinformatics & computational biology, cardiac allograft vasculopathy (CAV), macrophage - cell, transplantation (heart)

## Abstract

Chronic allograft vasculopathy (CAV) is a major cause of late graft failure in heart transplant recipients, characterized by progressive intimal thickening and diffuse narrowing of the coronary arteries. Unlike atherosclerosis, CAV exhibits a distinct cellular composition and lesion distribution, yet its pathogenesis remains incompletely understood. A major challenge in CAV research has been the limited application of advanced “-omics” technologies, which have revolutionized the study of other vascular diseases. Recent advancements in single-cell and spatial transcriptomics, proteomics, and metabolomics have begun to uncover the complex immune-endothelial-stromal interactions driving CAV progression. Notably, single-cell RNA sequencing has identified previously unrecognized immune cell populations and signaling pathways implicated in endothelial injury and vascular remodeling after heart transplantation. Despite these breakthroughs, studies applying these technologies to CAV remain sparse, limiting the translation of these insights into clinical practice. This review aims to bridge this gap by summarizing recent findings from single-cell and multi-omic approaches, highlighting key discoveries, and discussing their implications for understanding CAV pathogenesis.

## Introduction

Atherosclerosis, characterized by the progressive buildup of plaques within arterial walls, is a leading cause of ischemic heart disease and heart failure. In severe cases where cardiac function is irreversibly compromised, heart transplantation remains the only viable option to fully restore cardiac function and improve patient survival. In the US alone, over 3,000 heart transplantations are performed annually (1). However, approximately 30% of cardiac allografts fail within the ten years of transplantation, with a significant proportion attributed to cardiac allograft vasculopathy (CAV) after 3 years following transplantation (2–4) CAV is a progressive, intimal hyperplastic lesion affecting both arteries and veins, leading to maladaptive vessel narrowing that clinically presents in a wide range of pathologies including myocardial infarction and sudden cardiac death.

CAV shares several similarities with atherosclerosis but also exhibits notable distinctions. Unlike atherosclerosis, which often presents as focal, eccentric lesions, CAV is a diffuse, concentric process that involves the entire coronary vascular tree (5). While atherosclerosis is a long-life remodeling process starting in childhood and manifesting in advanced adult age, CAV exhibits a rapid onset within months after transplantation. The rapid onset of CAV is triggered by immune-mediated processes initiated by alloimmune responses, while atherosclerosis is primarily driven by prolonged lipid accumulation and metabolic stress that leads to endothelial dysfunction. Even though the progression of disease pathology is distinct, both atherosclerosis and CAV are mediated by both the innate and adaptive immune system, particularly macrophages, DCs, T cells, and B cells (6).

Pathologically, CAV is predominantly immune-mediated and characterized by intimal hyperplasia composed of accumulation of vascular smooth muscle cells (VSMCs), an intact internal elastic lamina, a preserved tunica media, and mononuclear cell infiltrations (5, 7). Compared to atherosclerotic regions, CAV lesions frequently feature intraplaque hemorrhages which may accelerate lesion progression and contribute to lumen stenosis (8). These pathological differences also influence treatment strategies. While atherosclerosis is managed primarily through a combination of lipid-lowering therapies (e.g., statins, PCSK9 inhibitors) (9) and anti-inflammatory agents, CAV requires immunosuppressive strategies such as mTOR inhibitors (sirolimus and everolimus) (10, 11), calcineurin inhibitors (tacrolimus) (12), and proliferation inhibitors (mycophenolate) (13). Additionally, the diffuse nature of CAV makes revascularization techniques like stenting or bypass surgery less effective compared to their use in atherosclerosis (14). Given the “unnatural” process of heart transplantation, understanding the molecular and cellular mechanisms driving the pathogenesis of CAV may provide valuable insights into the interplay between the immune system and the vasculature that may also underlie other vascular diseases.

The rapid advances in “-omics” methodologies have revolutionized our ability to investigate biology across space and at numerous molecular levels, encompassing DNA, RNA, proteins, and metabolites (15, 16). These approaches are increasingly being applied to the field of allograft pathology and CAV, offering new avenues to investigate the driving factors for disease progression (17–19) High-resolution multi-omics approaches provide unparalleled insights into the intricate tissue microenvironments that shape transplant outcomes, enabling a deeper understanding of the dynamic interactions between host and graft and the mechanisms driving alloimmune responses. These breakthroughs have the potential to redefine strategies for diagnosing, monitoring, and treating CAV, facilitating the development of precision medicine approaches in transplantation care (16). This review seeks to synthesize recent findings from a diverse array of bioinformatic approaches that have significantly advanced our understanding of immune-mediated processes underlying CAV (Table 1). Additionally, it highlights unresolved questions raised by these studies, identifying knowledge gaps and suggesting priority areas for future research to further elucidate CAV pathophysiology and improve prolonged clinical outcomes for patients with heart transplantation.

**Table 1:** Key computational biology studies to interrogate immune cell function during cardiac allograft vasculopathy and chronic rejection.

Specimen	Computational Approach	Immune cells profiled	Conclusions	Ref no.
Human - Allograft Explant Tissue	Single-nuclear RNA Transcriptomics	Macrophages, NK cells	• Donor-derived macrophages are replaced by recipient-derived macrophages • Recipient-derived macrophages are more fibrogenic and activate NK cells in allografts with CAV	(19)
Human – Allograft Explant Tissue	Spatial Transcriptomics	Macrophages, T cells, B cells	• CAV lesions in patients with DSA with low neointima had higher expression of inflammatory genes • CAV lesions with high neointima had more fibrotic profiles	(17)
Human – Coronary artery tissue & Blood	TCR/BCR Sequencing	T cells, B cells	• TCR sequencing revealed similar repertoire between blood and allograft, indicating an active bystander T cell response during CAV • Immunoglobulin heavy chain repertoire (B Cells) vary greatly from blood to allograft	(116, 98)
Human – Blood	Single-cell RNA Transcriptomics	Monocytes, T cells	• One of the first studies to characterize single-cell transcriptomes in PBMCs from heart transplant patients with or without CAV • Increased circulating CD14 + and CD16 + monocytes and CD4+ T memory cells	(45)
Human – Urine	Proteomics	–	• Proteomic analysis of urine measured a number of differentially expressed peptides in patients with CAV • Since CAV is difficult to observe and diagnose, urine biomarkers may be an easy way to predict CAV development/progression	(135)
Human – Blood	Proteomics	–	• Proteomics of circulating proteins identified CLEC4C, a marker of plasmacytoid DCs, as correlative to allograft dysfunction	(134, 148)
Human/Pig- Heart Xenografts	Bulk and Single-cell Transcriptomics, Lipidomics, Proteomics, and Metabolomics	NK Cells, T cells	• Multi-omics data shows rapid decline of xenograft due to increased CD8+ T cell infiltration into xenograft • Increased cold ischemic time correlated with increased presence of macrophages, neutrophils, and memory T cells.	(85)
Human – Heart, Lung, Liver, & Kidney Blood & Biopsies	Microarray, Bulk and Single-cell RNA Transcriptomics	Macrophages, T cells	• Identified conserved gene expression signatures across four solid organ transplantation datasets to profile ischemia reperfusion injury, acute rejection, fibrosis, and tolerance • Measured increased CD16 + monocyte/macrophage in rejecting heart transplants spanning over 150 datasets	(18)

### Immunity during cardiac allograft vasculopathy

One of the primary challenges in treating CAV is its delayed clinical presentation. Due to the lack of sensory nerves in the transplanted heart, CAV develops silently, without the typical warning signs of angina pectoris (20). As a result, it often manifests at an advanced stage presenting with symptoms of graft dysfunction, arrhythmias, or even sudden cardiac death. Although the clinical presentation of CAV typically emerge years after transplantation, early inflammatory events within the first year are critical to its initiation and pathogenesis (21). The upregulation of Human Leukocyte Antigen (HLA) class II molecules has been identified as a significant trigger of CAV in large cohorts of European and American heart transplant recipients (22). Additionally, acute cellular rejection, primarily mediated by T cells, has been recognized as an independent risk factor for CAV progression (22). While the precise mechanisms linking these inflammatory triggers to disease progression remain largely correlative, recent advances in large-scale biological approaches have greatly enhanced our understanding of CAV in preclinical models. In the following sections, we review the contributions of both the innate and adaptive immune systems to CAV progression. Future studies should aim to validate these findings in clinical cohorts and develop biomarkers or other diagnostic methods to improve early detection and intervention of CAV.

#### Monocytes and macrophages

Macrophages represent a heterogenous population within the innate immune system, performing diverse functions during homeostasis (23) and pathological conditions such as ischemia-reperfusion injury (24, 25), acute rejection (26, 27), and chronic rejection (28, 29) in cardiac transplantation. Macrophages play critical roles through the lifespan of an allograft, significantly influencing the onset and progression of CAV. Beyond their interactions with the adaptive immune system, macrophages actively shape the vasculature's microenvironment by secreting signals that activate fibroblasts and VSMCs driving fibrous intimal thickening of CAV (28, 30). Experimental depletion of myeloid cells after heterotopic heart transplantation suppresses the development of vasculopathy, underscoring their necessity in CAV pathogenesis (29, 31). Recent advances in single-cell and spatial transcriptomics technologies are beginning to unravel the intricate contributions of macrophages to CAV pathophysiology, shedding light on their origins, functional specialization, and localization within the heart.

Historically, macrophages were thought to originate exclusively from circulating monocytes, as described by the mononuclear phagocyte system. However, advanced genetic mouse models tracing the fates of myeloid precursor cells have demonstrated that tissue macrophages arise from distinct developmental origins, including macrophages residing within the heart (32–35) During fetal development, progenitors from the yolk sac and fetal liver seed tissues, giving rise to local resident macrophage populations that perform specialized roles within tissue-specific niches (36, 37). These yolk sac-derived resident macrophages, despite adapting to diverse tissue environments, exhibit high transcriptional conservation and are characterized by the expression of TIM-4, LYVE-1, or FOLR2 (38). Within the heart, these embryonically derived macrophages are able to self-renew, persisting well into adulthood (39). Throughout aging, however, resident cardiac macrophages are continuously replaced by monocyte-derived macrophages, which can be identified by the expression of C-C chemokine receptor 2 (CCR2) (34, 40).

**Figure 1:**
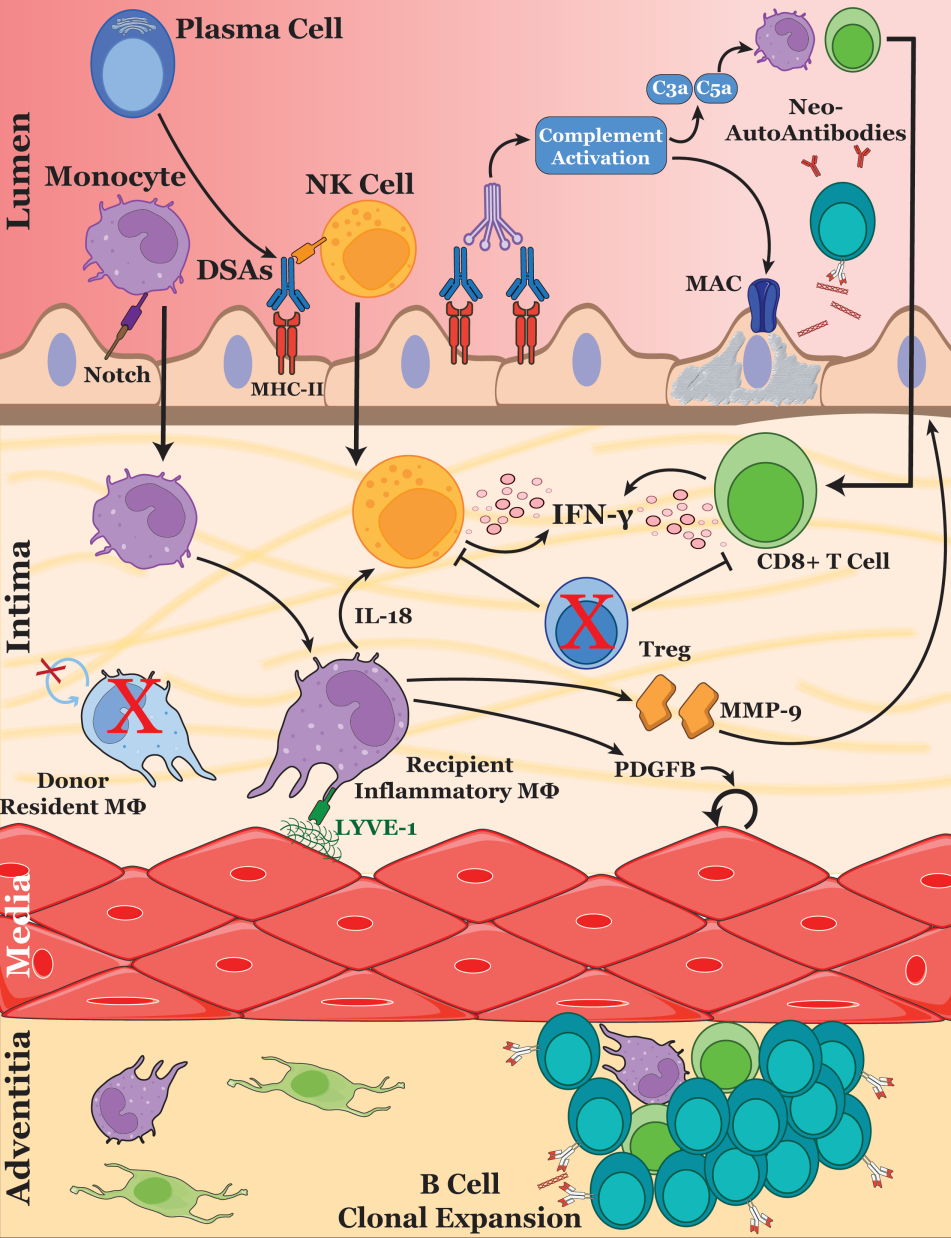
Immune cell interactions driving cardiac allograft vasculopathy. The schematic illustrates the key cellular and molecular mechanisms involved in the progression of CAV. After ischemic-reperfusion injury during heart transplantation, endothelial cell activation and the development of *de novo* DSAs promotes the recruitment of monocytes, NK cells and T cells into the inflamed vessel. Monocyte-derived macrophages are stimulated through Notch signaling by the endothelium, polarizing them into an inflammatory phenotype. Upon Lyve-1 stimulation, macrophages secrete MMP-9, remodeling the basement membrane and promoting further immune cell extravasation. Additionally, macrophages produce growth factors such as PDGF which drive VSMC proliferation—a hallmark of CAV. DSAs also promote complement activation, leading to the formation of the membrane attack complex and causing endothelial cell damage. Released DAMPs generate new autoantibodies, further amplifying antibody-mediated rejection and allograft injury. Regulatory T cells may play a protective role within the vessel by suppressing NK cell and CD8+ T cell activation. These cellular interactions collectively contribute to the remodeling of the vascular intima, leading to luminal narrowing and eventual graft dysfunction.

Insights from lineage-tracing studies and transplantation models have informed one another about macrophage origins and their immunogenic roles. Kory Lavine and colleagues reported that in sex-mismatched heart transplant recipients (female donors to male recipients), CCR2- macrophages lacked expression of the Y chromosome, demonstrating their donor tissue-residence origin (41). CCR2 + macrophages, on the other hand, represented a heterogeneous population containing cells both with and without Y chromosome expression, highlighting the significant contribution of peripheral monocyte recruitment to this macrophage subset in the human heart (41). Newer analyses of single cell transcriptomics now enable the differentiation of donor and recipient cells based on naturally occurring small-nucleotide variances (SNVs) (42, 43). In pediatric heart transplant recipients with CAV, almost 90% of myeloid cells within the allograft were of recipient origin by five days post-transplantation, with all cardiac immune cells transitioning to recipient origin by 15 months post-transplantation (19). This pattern mirrored observations in murine models of heterotopic heart transplantation (44). Shortly after transplantation, circulating monocytes from the recipient rapidly infiltrate the heart and polarize into proinflammatory macrophages which have been associated with CAV severity (Figure 1) (19, 45). The mechanisms by which this initial wave of inflammation, driven by this distinct population of hyperinflammatory macrophages, primes the vasculature for the chronic development of CAV remain incompletely understood. Additional epigenomic and transcriptomic studies are needed to elucidate the lasting effects of these macrophages on the vasculature and their role in the progression of CAV.

Importantly, donor-derived CCR2 + macrophages have been implicated in graft survival. Loss of these macrophages was associated with prolonged allograft survival, likely due to reduced signaling through the MyD88 pathway (44). This pathway plays a pivotal role in enabling antigen-presenting cells to present antigens to T cells, and its inhibition significantly extends graft survival. In contrast, selective depletion of donor CCR2- macrophages prior to transplantation has been shown to acutely reduce allograft survival (44). Therefore, CCR2- macrophages likely play a protective role early post-transplantation and their ensuing loss may result from process linked to allograft rejection. In human transplant recipients, immune suppression therapy appears to impair the proliferative capacity of donor CCR2- macrophages, thus leading to their eventual replacement by recipient monocyte-derived macrophages.

While recipient macrophages infiltrating the allograft exhibit transcriptomic similarities to donor-resident macrophages, they do not fully replicate their functional properties. Specifically, recipient-derived macrophages demonstrate reduced phagocytic activity yet an increased expression of inflammation-resolving genes (19). Natural defects in macrophage phagocytosis have been correlated with poor post-transplant outcomes, although a direct relation has yet to be fully established (46). Efficient clearance of dead cells by macrophages prevents the accumulation of immunogenic self-antigens and promotes tissue-reparative and tolerogenic signaling (47). Leveraging this tolerogenic function, interventions such as the injection of apoptotic donor cells have been shown to induce immune tolerance and prolong heart allograft survival in murine models (48–50) Notably, these recipient macrophages also exhibit enhanced fibrotic signaling, including elevated expression of PDGFB, a gene implicated in fibrotic remodeling of the heart (19, 51, 52). Bone marrow-derived myeloid cells upregulate PDGFB to also promote the proliferation of vascular smooth muscle cells, a process that is dependent on the efferocytosis receptor AXL (28). Further studies are needed to delineate the unique roles of donor- and recipient-derived CCR2- macrophages in apoptotic cell clearance, inflammation resolution, and fibrotic signaling during chronic rejection and CAV.

While these single cell approaches utilizing endomyocardial biopsies have tremendously increased our understanding of macrophage origin and function in the allografted heart, they may not fully capture the immune and vascular dynamics contributing to neointima formation. During homeostasis, two different interstitial macrophage populations exist across tissues including the heart, fat, dermis, and lung: one that preferentially surrounds the nerves and one that localizes with blood vessels (53). After cardiac transplantation, macrophages are present within the neointima and adventitia of CAV lesions and are associated with antibody-mediated rejection (54–56) Leveraging the utility of spatial transcriptomics and proteomics, Elaine Reed and colleagues revealed that arterial lesions with low neointimal thickness exhibit higher inflammatory and cell death signatures, whereas lesions with high neointimal thickness display remodeling and fibrotic profiles aligning with neointima expansion (17). These findings support the observations that inflammation precedes the expansion of the neointima through SMC accumulation followed by subsequent vascular fibrosis (57). Furthermore, macrophages around blood vessels express high levels of the hyaluronan receptor LYVE-1, which interacts with hyaluronic acid on the surface of VSMCs to promote collagen degradation through pericellular MMP-9 activity (58). Interestingly, in CAV-affected lesions, macrophages secrete MMP-9 in response to endothelial cells activated by anti-HLA class I donor-specific antibodies (59). MMP-9-producing monocytes degradation of the collagen basement membrane allows for invasion of T cells in large vessels (60). These findings highlight the complex interplay between macrophages, vascular smooth muscle cells, and immune responses in driving the progression of CAV, underscoring the need for further research to fully elucidate the mechanisms contributing to neointima formation and vascular fibrosis after transplantation.

#### Natural killer cells

Natural killer (NK) cells, lymphocytes of the innate immune system, have been implicated in the development and progression of transplant-associated arteriosclerosis (61). NK cells control pathogenic viral infections and eliminate malignant cells, but their role extends beyond cytotoxicity as they also secrete cytokines and chemokines, most notably IFN-*γ* (62–64) Historically, NK cells were defined by their morphology and function of that of a large granular lymphocyte that can *kill* its target cells *naturally*, meaning NK cells were not restricted by MHC expression on the target cell (65, 66). These insights stemmed from the peculiar observations by Gustavo Cudkowicz and Michael Bennet that F1 hybrid mice reject the transplantation of parental bone marrow cells (67, 68) leading to the “missing self-hypothesis” which states that NK cells would eliminate target cells that lacked self MHC-I molecule expression (69). Further studies revealed that NK cells expressed killer immunoglobulin-like receptors (KIRs) that engage MHC-I molecules to generate an inhibitory signal to prevent NK-mediated cell killing (70). In the context of HLA class I mismatches in transplantation, NK cells are likely to perceive graft cells as “missing self”, triggering NK cell activation and subsequent endothelial cell death (71). Further supporting this hypothesis, it was demonstrated that in semi-allogenic cardiac transplants between parental donors and F1 hybrid recipients, that NK cells contribute to the development of CAV by recruiting T cells through secretion of IFN-*γ* (72). Remarkably, even in the absence of T and B cells, NK cells are sufficient to promote vasculopathy in mice (73). This phenomenon may stem from their role in viral immunity, as lymphocytic choriomeningitis virus can induce CAV through NK cell activity without involvement of adaptive immune cells (74). Moreover, NK cell inhibition in T-cell-depleted mice resulted in prolonged acceptance of cardiac allografts (75). Together, these findings reveal that NK cells are key drivers of chronic vascular injury, significantly contributing to the development and progression of allograft vasculopathy.

NK cells efficiently lyse target cells without prior stimulation, relying on a finely tuned balance of activating and inhibitory signals mediated by their receptors (66). Inhibition of one such activating receptor, NKG2D, has been shown to prolong the survival of allografted hearts (76, 77). However, contradictory findings suggest that NKG2D deletion can also accelerate heart allograft rejection (78). These contrasting outcomes highlight the duality of NK cell functions: while they are associated with chronic graft injury, they can also promote tolerance by targeting donor antigen-presenting cells (APCs) and preventing their migration to recipient secondary lymphoid tissue (79). However, this tolerogenic role of NK cells is likely diminished as donor APCs are rapidly replaced by recipient-derived cells (19). Beyond their direct cytotoxic effects, NK cells are integral to the immune response to donor-specific antibodies (DSAs). NK cell-mediated IFN-*γ* production and contact-dependent cytotoxic activity are rate-limiting effector pathways during antibody-induced chronic allograft vasculopathy (80). Through the Fc receptor CD16a, NK cells recognize DSAs, triggering antibody-dependent cytotoxicity (81). Notably, elevated CD16a expression is linked to a higher risk of CAV in human patients, driving both IFN-*γ* production and the release of cytotoxic molecules (82, 83).

Recent advances in single-cell sequencing have revealed a distinct subset of NK cells that are linked to dysfunctional allografts and persistent CAV (19). These NK cells exhibit elevated levels of IFN-*γ*, CRTAM, and Fas-ligand, linking them to chronic inflammatory and cytotoxic processes within the graft. Further analysis of cell communication from single-cell data suggests that proinflammatory macrophages regulate NK cell activation via IL-18 and CSF2 signaling (19, 84). This interaction underscores the adaptability of NK cells and their significant role in long-term graft dysfunction. In human decedent studies with pig heart xenografts, integrative multi-omics analysis showed a marked increase in NK cell activity as early as one day after transplantation (85). Secreted IFN-*γ* by NK cells promotes the production of CXCL9 and CXCL10 by cardiac fibroblasts, essential chemokines for NK cell recruitment, thereby creating a persistent feedforward loop within the allograft (86, 87). Furthermore, IFN-*γ* has long been recognized as a central effector in allograft arteriosclerosis due to its pleiotropic roles in cell proliferation, death, inflammation, and fibrosis (88). Together, these findings highlight a complex and self-sustaining network of NK cell activation and chemokine signaling within dysfunctional allografts, emphasizing the need for targeted therapeutic strategies to disrupt this feedforward loop and mitigate chronic graft inflammation and dysfunction.

#### B cells and antibodies

The innate immune system alone is not sufficient for causing chronic rejection of cardiac allografts (72, 89). The interplay between adaptive immune cells and antibody-mediated mechanisms is now recognized as a significant contributor to the progression of allograft vasculopathy within patients, particularly with the strong association of DSAs to chronic allograft rejection and CAV (90). Early studies demonstrated that T cells are necessary for CAV development as transfer of T cells in immunodeficient mice successfully recapitulated the characteristic vascular damage of CAV (91). In contrast, the role of B cells in CAV has been more uncertain, with findings varying based on mouse models and the immunosuppressive regimens used to induce CAV (92–95) Despite these challenges in animal models, clinical evidence shows a clear association between the presence of B and plasma cells around coronary arteries in patients with CAV—a feature that distinguishes it from atherosclerosis (96, 97). Additionally, analysis of clonal expansion of B cells using next generation sequencing of single cell methods revealed that specific B cell clones undergo robust expansion within the allograft (98).

These B and plasma cells, along with T cells and macrophages, are frequently organized into tertiary lymphoid-like structures within the adventitia (97, 99). While initially hypothesized to be a local source of DSAs, these nodules of B cells were found to exhibit a polyreactive profile, with a majority of cells secreting natural antibodies that react to autoantigens (100). One such autoantigen is vimentin, an intermediate filament protein that is expressed within the cytosol of smooth muscle cells and fibroblasts, where antibodies against it have been predictive of vasculopathy in heart allografts (101, 102). Although the exact mechanism by which intracellular vimentin expression contributes to the production of anti-vimentin antibodies during CAV remains unclear, it has been shown that vimentin becomes a target of caspases during inflammation, leading to the exposure of antigenic vimentin in apoptotic cells (103). Further research is needed to elucidate the processing and presentation of vimentin, as well as other “self” targets, to B cells, driving the production of these autoantibodies.

The generation of *de novo* DSAs by B cells plays a critical role in the development and progression of CAV through antibody-mediated rejection mechanisms (Figure 1) (104). B cells residing within the grafted endothelium are actively generating DSAs against donor MHC-I molecules (105). These antibodies have long been linked to CAV, where the extent of HLA mismatches is associated with allograft rejection (106–108) Indeed, the transfer of DSAs targeting MHC-I has been shown to initiate endothelial inflammation followed by the development of CAV, independent of complement fixation that often accompanies acute antibody-mediated rejection (109, 110). In an intriguing murine model of CAV, CCR5-deficient and CD8-deficient mice, transiently treated with anti-CD4 therapy, developed an exaggerated antibody response after cardiac transplantation, leading to the generation of DSAs and subsequent vasculopathy (56). Notably, variability in DSA titers within this model closely correlated with the severity of vasculopathy, reflecting similar observations in human heart transplantation patients (56, 111). Thus, the dual role of B cells in producing both donor-specific and self-reactive antibodies significantly contributes to endothelial injury, amplifies immune responses, and drives the development of chronic allograft vasculopathy.

Antibodies play a multifaceted role in activating immune responses during allograft rejection. One of the most well defined roles of antibodies is their ability to activate the classical pathway of the complement cascade, synergizing their direct effects on immune cells during vasculopathy (96). Upon binding to antibodies, C1 activates the complement cascade by cleaving C4 and C2, forming the C4b2a complex, also known as C3 convertase. This enzyme cleaves C3 into two fragments, C3a and C3b. C3b is a chief component of the complement system as it can readily coat pathogens to promote their clearance, combine with other components of the complement system to form the membrane attack complex (MAC), and initiate a self-perpetuating amplification loop to produce more C3b (96). Additionally, many of the cleavage products of the complement system serve as chemoattractant signals to recruit neutrophils, monocytes, NK cells, B cells, and T cells to the vessel.

Antibodies also exert direct effects on endothelial and smooth muscle cells within the coronary arteries. DSAs for HLA class I antigens modulate endothelial cell function by stimulating the release of von Willebrand factor and P-selectin, leading to the aggregation of platelets and recruitment of circulating monocytes (112). Engagement of MHC class I molecules by antibodies increased expression of fibroblast growth factor receptor enhancing the proliferative responses of vascular smooth muscle cells (113). Furthermore, clinical biopsies of cardiac allografts undergoing antibody-mediated rejection and mouse models with high titers of DSA revealed upregulation of the Notch ligand *Dll4*, specifically with the lesions of large arteries (56, 114). Interestingly, endothelial *Dll4* induces inflammatory polarization of macrophages leading to their production of the inflammatory cytokine IL-6 (114). Lastly, many immune cells including macrophages and NK cells contain receptors that recognize the Fc region of antibodies (Figure 1). Engagement of the Fc*γ*RIII(CD16a) receptor on NK cells initiates antibody-dependent cell-mediated cytotoxicity on the endothelium (82). Endothelial cell death may further diversify the antigenic load of the allograft (83), possibly leading to the production of non-DSA autoantigens such as vimentin.

In summary, antibodies orchestrate a complex and multifaceted immune response during allograft rejection by activating the complement cascade, recruiting and modulating immune cells, directly influencing vascular cells, and promoting inflammatory and cytotoxic pathways. These diverse mechanisms underscore the central role of antibodies in driving vasculopathy and immune-mediated injury, ultimately contributing to allograft dysfunction and failure.

#### T cells

T cells are key players in cellular-mediated rejection, which is often associated with acute rejection, but their contributions to chronic rejection and allograft vasculopathy remain less clearly understood. T cells do contribute to chronic rejection as mice lacking an adaptive immune system fail to reject cardiac allografts until activated T cells are transferred exogenously (89). In humans, both CD4 + and CD8+ T cells infiltrated the intima and adventitia of large coronary arteries associated with allograft vasculopathy (115). In the absence of CD4+ T cells, primed CD8+ T cells were sufficient to develop robust CAV (91). Deletion of effector molecules within these CD8+ T cells revealed that these cells perform a distinct IFN-*γ*-dependent mechanism to promote vasculopathy along with direct cytolysis. During acute rejection of cardiac allografts, TCR sequencing reveals a robust expansion of cytotoxic CD8+ T cell clones (86). However, in patients with an HLA-mismatched graft and who developed vasculopathy, the T cell repertoire did not differ significantly between the circulating blood and grafted tissue indicating a lack of clonal expansion within the allograft (45, 116). In contrast, the repertoire of BCR sequences with the graft minimally overlapped with the circulating B cells providing further evidence that chronic rejection may be driven more by B cell expansion (98).

While T cells may now be considered “bystanders” in the immune processes driving chronic vasculopathy, they contribute significantly to the inflammatory environment within the vasculature, even in the absence of antigen specificity. Infiltrating T cells are key producers of IFN-*γ* and TGF-*β*, two critical mediators of inflammation and fibrosis that drive the pathophysiology of CAV (116, 117). Additionally, endothelial cells upregulate nitric oxide signaling in bystander CD8+ T cells via iNOS expression, a process linked to vascular dysfunction (118, 119). In contrast, regulatory T cells (Tregs) are a specialized subset of T cells that play a critical role in maintaining immune homeostasis by suppressing excessive immune responses and promoting tolerance to self and non-self-antigens. Although Tregs are not detected in large numbers within coronary arteries with CAV lesions (117), their expansion within cardiac allografts has been shown to attenuate CAV progression and delay chronic rejection (120–122) Conversely, depletion of Tregs leads to uncontrolled activation of NK cells, which accelerates CAV progression (123, 124). Interestingly, strategies that block memory T cell activation or deplete gamma delta T cells have been shown to promote Treg expansion (124, 125). Together, these findings illustrate that distinct T cell populations play active and opposing roles in shaping the inflammatory and fibrotic environment that drives the progression of CAV (Figure 1).

### Translating bioinformatics into clinical practice

#### Applications

The rapid advancements in single-cell sequencing, spatial transcriptomics, proteomics, metabolomics, and other bioinformatic techniques have revolutionized our understanding of the molecular and cellular mechanisms underlying the development of CAV in heart transplant patients (Table 1). These technologies have not only deepened our knowledge of disease progression in humans but also enhanced our ability to refine animal models, improving the gap between preclinical studies and human pathology. Importantly, as these techniques continue to evolve, they bring us closer to their application in clinical practice. Ultimately, these powerful tools will transform patient care by enabling more accurate prediction, early diagnosis, and personalized management of rejection and vasculopathy.

Gene expression profiling has long been recognized as a powerful tool for non-invasive prediction of cardiac vasculopathy, offering an alternative to the more invasive endomyocardial biopsy. By analyzing gene expression patterns in transplant patients' blood across multiple centers, researchers identified a set of genes optimized for detecting acute allograft rejection (126). This discovery led to the development of AlloMap, a clinical tool that non-invasively predicts acute cellular rejection by profiling the expression of these specific genes. While AlloMap has demonstrated a strong negative predictive value for ruling out rejection, its positive predictive value is limited, particularly in predicting CAV, as it was specifically designed for acute cellular rejection surveillance (127). More recent advancements in gene expression analysis have identified rejection-associated transcripts that include many key molecular mediators of immunity discussed in the preceding sections of this review. These rejection-associated transcripts have shown improved utility in diagnosing antibody-mediated rejection from endomyocardial biopsies, offering a more precise tool for detecting rejection and CAV (128, 129). Beyond traditional transcriptomics, emerging technologies like spatial transcriptomics hold immense promise for advancing clinical care (130). This cutting-edge platform allows for the spatial visualization of gene expression within specific structures, such as coronary arteries, enabling direct assessment of pathological lesions from biopsies. Though still in its infancy, particularly in heart transplantation, spatial transcriptomics has the potential to revolutionize the diagnosis and treatment of CAV by providing unparalleled insight into the molecular and spatial landscape of the disease. As these technologies mature, they promise to redefine the standard of care for transplant patients, paving the way for more precise, personalized, and effective interventions.

A unique and innovative application of sequencing technologies in transplantation is the measurement of donor-derived cell-free DNA (dd-cfDNA) in the blood of recipients. This biomarker reflects tissue damage, as cell-free DNA is released from damaged donor cells into the recipient's bloodstream. Initially utilized to predict acute rejection after heart transplantation, elevated dd-cfDNA levels have been strongly associated with acute rejection episodes (131). In the context of chronic rejection, higher dd-cfDNA levels in patients with CAV have been linked to the presence of *de novo* DSAs and the development of vasculopathy (132, 133). Despite these promising associations, the relationship between dd-cfDNA levels and the severity of CAV remains unclear and warrants further investigation. Overall, measuring dd-cfDNA has become a critical tool in the clinical management of solid organ transplantation, offering a non-invasive method to monitor graft health and predict rejection.

The widespread and deep profiling of other biomolecules, including proteins and metabolites, offer significant potential for improving the clinical management of heart transplant patients. Proteomics has been employed to identify novel biomarkers associated with an increased risk of post-graft dysfunction (134). Additionally, urinary proteomic signatures are a potential non-invasive tool for the surveillance of CAV progression (135). Similarly, metabolomics is gaining traction in transplantation research. For instance, studies have shown that ex vivo perfusion can normalize the metabolomes of hearts procured from deceased cardiac donors and those from brain-dead donors, potentially improving organ viability and function prior to transplantation (136). While the development of these tools may be lagging behind the translation of transcriptomics into clinical practice, these advancements underscore the promise of proteomics, metabolomics, and other “-omics” in accelerating the discovery of biomarkers that will enhance the diagnostic and prognostic strategies after heart transplantation.

#### Challenges and future directions

The promise and application of bioinformatics at both the bench and bedside are advancing rapidly, revolutionizing our understanding of chronic allograft vasculopathy. However, it is essential to recognize their current limitations and how these constraints may affect their clinical and research applications. High throughput approaches require the processing of a large quantity of data, which requires advanced and rigorous analyses to determine the underlying biological information. Proteomic and metabolomic tools that rely on mass spectrometry for biomolecule quantification face significant challenges in standardization and sensitivity, which hinder their translation into clinical diagnostic tests (137). For instance, the metabolome is highly sensitive to environmental and technical factors, making it difficult to achieve consistent results across clinical laboratories. Additionally, variation among samples processed through the same mass spectrometry pipeline can exceed biological differences, complicating the identification of true metabolic changes (138, 139). Overcoming these obstacles remains a significant challenge for the future application of these mass spectrometry-based technologies in both the clinical and research setting.

Single-cell RNA sequencing, while a powerful tool for delineating cell-specific gene expression, is subject to inherent biases such as dropout events, low RNA capture efficiency, and batch effects, all of which can confound downstream analyses (140–142). For instance, the high cycle numbers required for polymerase chain reaction amplification can reduce the detection and analysis of low-abundance RNA transcripts, potentially obscuring biologically significant signals. However, rapidly evolving spatial transcriptomic and single cell sequencing methodologies are enhancing the sensitivity and resolution of these technologies, enabling the capture of greater transcriptomic complexity within individual cells (143). Despite these advancements, one of the most significant challenges in large-scale biological data analysis is the presence of unwanted variation, specifically “batch effects”, which represent variation originating from technical differences across samples that are unrelated to the biological variables being studied. Excitingly, new computational tools continue to expand the bioinformatician's toolbox, with methods such as Harmony (144) and Seurat (145) offering powerful approaches to mitigate batch effects while preserving true biological variance, ultimately improving the robustness and interpretability of single-cell analyses (146).

The most successful emerging application of large-scale biological data within the field of transplantation is the ability to measure dd-cfDNA using a blood test, however there are significant limitations of this approach. Initially sought to be a specific marker of allograft rejection, dd-cfDNA is more indicative of allograft tissue injury and cannot necessarily distinguish between acute or chronic rejection. Furthermore, economic analyses suggest that the cost of biomarkers, such as dd-cfDNA, may actually be less cost effective than the typical screening with protocol biopsy (147). As bioinformatics and “-omics” technologies continue to evolve, their integration into clinical transplantation has the potential to revolutionize diagnostics and patient management. However, overcoming technical challenges such as standardization, sensitivity, and cost-effectiveness remains crucial to ensuring their successful translation from research to routine clinical practice.

## Conclusion

The application of computational approaches, such as single-cell sequencing, spatial transcriptomics, proteomics, and metabolomics, has significantly enhanced our understanding of chronic allograft vasculopathy and its complex pathophysiology. These tools have provided unparalleled insights into the molecular mechanisms driving CAV, including the intricate immune-endothelial interactions and novel cellular populations contributing to endothelial dysfunction and intimal thickening. Understanding the immune landscape of CAV, particularly the roles of macrophages, NK cells, T cells, and antibodies, will be critical for developing targeted therapies to enhance graft survival and mitigate chronic rejection. Additionally, they have led to the identification of new biomarkers that could improve early diagnosis, prognosis, and monitoring for vasculopathy, a typically challenging diagnosis requiring invasive imaging. Despite the promising advances, the clinical application of these technologies is still in its early stages. However, the potential to transform the diagnosis, treatment, and prevention of CAV is substantial. As these technologies become more refined, accessible, and affordable, they could revolutionize how we manage transplant patients. As these technologies grow more complex in both methodology and analysis, developing standardized platforms will be crucial to streamline workflows and enhance data interpretation in both research and clinical settings. By bridging the gap between molecular research and clinical practice, these innovations hold the potential to reshape CAV management, offering hope for more effective strategies that address chronic allograft rejection and improve the long-term survival of transplant recipients.
